# The risk-benefit task of research ethics committees: An evaluation of current approaches and the need to incorporate decision studies methods

**DOI:** 10.1186/1472-6939-13-6

**Published:** 2012-04-20

**Authors:** Rosemarie D L C Bernabe, Ghislaine J M W van Thiel, Jan A M Raaijmakers, Johannes J M van Delden

**Affiliations:** 1 Julius Center for Health Sciences and Primary Care, Utrecht University Medical Center, Heidelberglaan 100, Utrecht, 3584CX, the Netherlands; 2 GlaxoSmithKline, Huis ter Heideweg 62, Zeist, 3705 LZ, the Netherlands

**Keywords:** Risk benefit assessment, Ethics committee, IRB, Decision theory, Net risk test, Component analysis

## Abstract

**Background:**

Research ethics committees (RECs) are tasked to assess the risks and the benefits of a trial. Currently, two procedure-level approaches are predominant, the Net Risk Test and the Component Analysis.

**Discussion:**

By looking at decision studies, we see that both procedure-level approaches conflate the various risk-benefit tasks, i.e., risk-benefit assessment, risk-benefit evaluation, risk treatment, and decision making. This conflation makes the RECs’ risk-benefit task confusing, if not impossible. We further realize that RECs are not meant to do all the risk-benefit tasks; instead, RECs are meant to evaluate risks and benefits, appraise risk treatment suggestions, and make the final decision.

**Conclusion:**

As such, research ethics would benefit from looking beyond the procedure-level approaches and allowing disciplines like decision studies to be involved in the discourse on RECs’ risk-benefit task.

## Background

Research ethics committees (RECs) are tasked to do a risk-benefit assessment of proposed research with human subjects for at least two reasons: to verify the scientific/social validity of the research since an unscientific research is also an unethical research; and to ensure that the risks that the participants are exposed to are necessary, justified, and minimized [1].

Since 1979, specifically through the Belmont Report, the requirement for a “systematic, nonarbitrary analysis of risks and benefits” has been called for, though up to the present, commentaries about the lack of a generally acknowledged suitable risk-benefit assessment method continue [1]. The US National Bioethics Advisory Commission (US-NBAC), for example, stated the following in its 2001 report on *Ethical and Policy issues in Research Involving Human Participants:*

"An IRB’s^1^

An institutional review board (IRB) is synonymous to an ethics committee. For consistency’s sake, we shall use REC throughout this paper.

assessment of risks and potential benefits is central to determining that a research study is ethically acceptable and would protect participants, which is not an easy task, because there are no clear criteria for IRBs to use in judging whether the risks of research are reasonable in relation to what might be gained by the research participant or society [2]."

The lack of a universally accepted risk-benefit assessment criteria does not mean that the research ethics literature says nothing about it. Within this same 2001 report, the US-NBAC recommended Weijer and Miller’s Component Analysis to RECs in evaluating clinical researches. As a reaction to Weijer and P. Miller, Wendler and F. Miller proposed the Net Risk Test. For convenience sake, we shall use the term “procedure-level approaches” [3] to refer to the models of Weijer et al. and Wendler et al.

In spite of their ideological differences, both procedure-level approaches are procedural in the sense that both approaches propose a step-by-step process in doing the risk-benefit assessment. In this paper, we shall not tackle their differences; rather, we are more interested in their similarities. We are of the position that both approaches fall short of providing an evaluation procedure that is systematic and nonarbitrary precisely because they conflate the various risk-benefit tasks, i.e., risk-benefit analysis, risk-benefit evaluation, risk treatment, and decision making [4-6]. As such, we recommend clarifying what these individual tasks refer to, and to whom these tasks must go. Lastly, we shall assert that RECs would benefit by looking into the current inputs of decision studies on the various risk-benefit tasks.

## The procedure-level approaches

**Figure 1:**
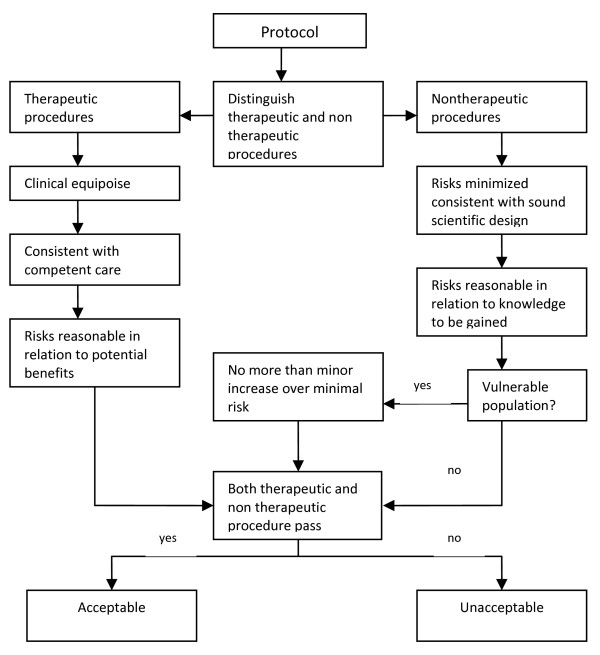
**Component Analysis **[7,9].

Charles Weijer and Paul Miller’s Component Analysis (Figure 1) requires research protocol procedures or “components” to be evaluated separately, since the probable benefits of one component must not be used to justify the risks that another component poses [2]. In this system, RECs would need to make a distinction between procedures in the protocol that are with and those that are without therapeutic warrant since therapeutic procedures would need to be analyzed differently compared to those that are non-therapeutic. It works on the assumption that a therapeutic warrant, that is, the reasonable belief that participants may directly benefit from a procedure, would justify more risks for the participants [7]. As such, therapeutic procedures ought to be evaluated based on the following conditions, in chronological order: that clinical equipoise exists, that is, that there is an “honest professional disagreement in the community of expert practitioners as to the preferred treatment” [8]; the “procedure is consistent with competent care; and risk is reasonable in relation to potential benefits to subjects” [7]. Non-therapeutic procedures, on the other hand, would need to be evaluated on the following conditions: the “risks are minimized and are consistent with sound scientific design; risks are reasonable in relation to knowledge to be gained; and if vulnerable population is involved, (there must be) no more than minor increase over minimal risk” [7]. Lastly, the REC would need to determine if both therapeutic and non-therapeutic procedures are acceptable [7]. If all components “pass”, then the “research risks are reasonable in relation to anticipated benefits” [7].

**Figure 2:**
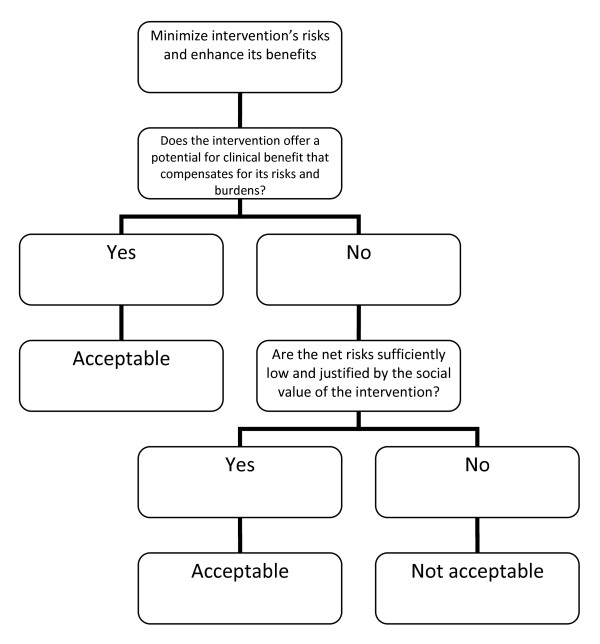
**The Net Risk Test **[10].

David Wendler and Franklin Miller, on the other hand, developed the Net-Risk Test (Figure 2) as a reaction to the Component Analysis. This system requires RECs to first “minimize the risks of all interventions included in the study” [10]. After which, the REC ought to review the remaining risks by first looking at each intervention in the study, and evaluating if the intervention “offers a potential for clinical benefit that compensates for its risks and burdens” [10]. If an intervention does offer a potential benefit that can compensate for the risks, then the intervention is acceptable; otherwise, the REC would need to determine whether the net risk is “sufficiently low and justified by the social value of the intervention” [10]. By net risk, they refer to the “risks of harm that are not, or not entirely, offset or outweighed by the potential clinical benefits for participants” [11]. If the net risks are sufficiently low and are justified by the social value of the intervention, then the intervention is acceptable; otherwise, it is not. Lastly, the REC would need to “calculate the cumulative net risks of all the interventions…and ensure that, taken together, the cumulative net risks are not excessive” [10].

**Figure 3:**
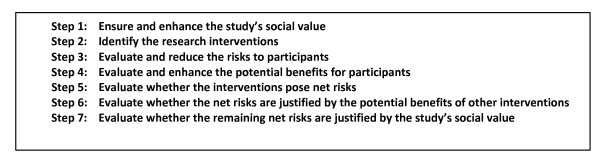
**Seven-step framework for risk-benefit evaluations in biomedical research **[11].

Recently, Rid and Wendler elaborated the Net Risk Test through a seven-step framework (see Figure 3) that is meant to offer a chronological, “systematic and comprehensive guidance” for the risk-benefit evaluations of RECs [11]. As we could see from Figure 3, most of the steps are the same as that of the previously explained Net Risk Test; the main addition of the framework is the first step, which is to ensure and enhance the study’s social value. In this first step, Rid and Wendler meant that RECs, at the start of their risk-benefit evaluation, ought to “ensure the study methods are sound”; “ensure that the study passes a minimum threshold of social value”; and “enhance the knowledge to be gained from the study” [11]. It is only after the social value of the study has been identified, evaluated, and enhanced could the RECs identify the individual interventions and then go through the other steps, i.e., the steps we have earlier discussed in the Net Risk Test.

## The procedure-level approaches and the conflation of risk-benefit analysis, risk-benefit evaluation, risk treatment, and decision making

These procedure-level approaches may be credited for providing some form of a framework for the risk-benefit assessment tasks of RECs. They have also provided RECs with a framework that includes and puts into perspective certain ethical concepts that may or may not have been considered in REC evaluations, but are now procedurally necessary concepts. Weijer and Miller, for example, made it necessary for RECs to always consider therapeutic warrant, equipoise, and minimal risk when evaluating the risk-benefit balance of a study. Wendler and Miller on the other hand, provided RECs with the concept of net risk. In spite of these contributions, these approaches presuppose (maybe unwittingly) that risk-benefit analysis, risk-benefit evaluation, risk treatment, and decision making can all be conflated. This, in our view, is a major error that ought to be corrected since from this error flow other problems, problems that unavoidably make the procedures unsystematic and arbitrary. To substantiate our view, we first have to make a necessary detour by discussing the distinction between risk-benefit analysis, risk-benefit evaluation, risk treatment, and decision making [4,5]. After which, we shall show how the conflation is present in the procedure-level approaches and how such a conflation leads to difficult problems.

### *Distinction between risk-benefit analysis, risk-benefit evaluation, risk treatment, and decision making*

Decisions on benefits and risks in fact involve four activities: risk-benefit analysis, risk-benefit evaluation, risk treatment, and decision making [4-6]. In the current debate, these terms are used as if they are interchangeable. Precisely because these four activities have four different demands, it must be made clear that the problem is not merely on terminological preference; that is, the problem cannot be solved by simply “agreeing” to use one term over another. In risk studies, the risk-benefit task concretely demands four separate activities [4,6]. Hence, these terms are not interchangeable, and their order must be chronological. The distinctions among these tasks and the necessity of their chronological ordering are as follows.

**Figure 4:**
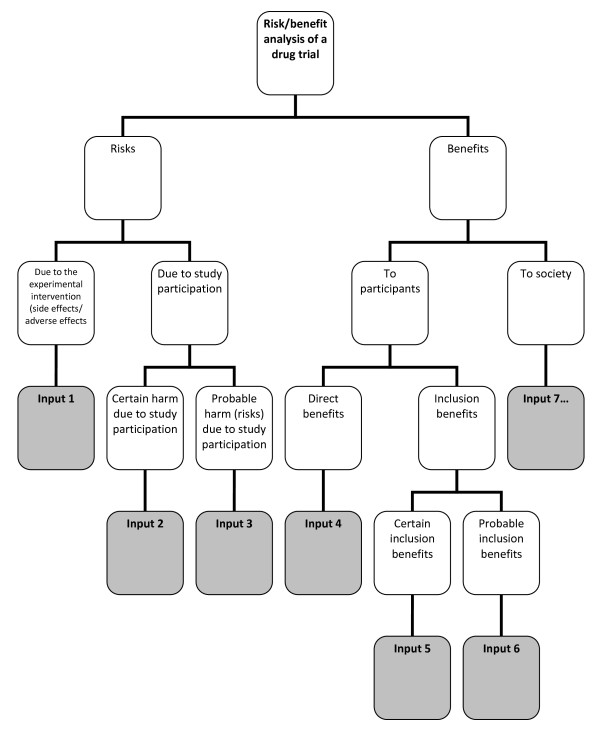
**Risk-benefit analysis value tree**.

*Risk-benefit analysis* refers to the “systematic use of information to identify initiating events, causes, and consequences of these initiating events, and express risk (and benefit)” [4]. This, risk-benefit analysis refers to 1.) gathering of risk and benefit events, causes, and consequences; and 2.) presenting this wealth of information in a systematic and comprehensive way, in accordance with the purpose why such information is systematized in the first place. There are a number of risk analysis methods such as fault tree analysis, event tree analysis, Bayesian networks, Monte Carlo simulation, and others [4]. The multi criteria decision analysis (MCDA) method, mentioned by the EU Committee for Medicinal Products for Human Use (CHMP) in the *Reflection Paper on Benefit Risk Assessment Methods in the Context of the Evaluation of Marketing Authorization Applications of Medicinal Products for Human Use*[12]*,* proposes the use of a value tree in analyzing the risk-benefit balance of a drug, for example. Adjusted to drug trials, a drug trial risk-benefit analysis value tree could look like (Figure 4).

In this value tree (Figure 4), we used King and Churchill’s typology of harms and benefits [1]. From each of the branches, the risk analyst would fill in information about a specific study. Of course, there could be more than one input under each category, depending on the nature of the drug trial being analyzed. Also, this value tree serves as an example; this is not the only way that benefits and risks may be analyzed within the context of drug trials. The best way to analyze risks and benefits within this context is something that ought to be further discussed and developed. Our aim is simply to show that a method such as a value tree is capable of encapsulating and framing the multidimensional nature of the causes and consequences of the benefits and risks of a study within one “tree.” This provides a functional risk-benefit picture from which the risks and the benefits may be evaluated, i.e., risk-benefit evaluation.

*Risk-benefit evaluation* refers to the “process of comparing risk (and benefit) against given risk (and benefit) criteria to determine the significance of the risk (and the benefit)” [4]. There are a number of methods to evaluate benefits and risks. Within the MCDA model for example, the “identification of the risk-benefit criteria; assessment of the performance of each option against the criteria; the assignment of weight to each criterion; and the calculation of the weighted scores at each level and the calculation of the overall weighted scores”[13] would constitute risk evaluation. The multriattribute utility theory (MAUT) is yet another example of an evaluation method. The MAUT is a theory that is basically “concerned with making tradoffs among different goals” [14]. This theory factors in human values, values defined as “the functions to use to assign utilities to outcomes” [14]. From the value tree “inputs,” the evaluator would then need to assign weights to each of these inputs. The purpose of plugging in weights is to establish the importance of each input, according to the evaluators. This is tantamount to establishing criteria, or identifying and making explicit the evaluators’ definition of acceptable risk. Next, the evaluators would need to plug in numerical values as the utility values of those that are being evaluated. These values would be multiplied to the weight. The latter values, when summed, would constitute the total utility value. To illustrate, if, for example, an REC wishes to make an evaluation of a psychotropic study drug and the standard drug, an REC may come up with MAUT chart like (Table 1).

**Table 1:** MAUT risk-benefit evaluation

	**Input 1:** **Grave adverse effects**	**Input 2:** **Trigger secondary psychological problems**	**Input 3:** **Suicidal ideation**	**Input 4:** **Safety with younger patients**	**Input 5:** **Dosage**	**Input 6:** **Administration…**	**TOTAL UTILITY**
Study drug	50	40	90	50	100	100	50 (1) + 40 (1) + 90 (.9) + 50 (.5) + 100 (.4) + 100 (.4) + …
Standard drug	70	80	40	100	100	50	70 (1) + 80 (1) + 40 (.9) + 100 (.5) + 50 (.4) + …
**WEIGHT**	1.0	1.0	.9	.5	.4	.4	

Just like the value tree, our purpose is not to endorse only one way of doing the evaluation. Our purpose is merely to illustrate that such a decision study tool is capable of explicitly showing the following: *a.)* the inputs that the evaluators think must play a role in the evaluation; *b.)* the values of the evaluators, through the scores they have provided; *c.)* the importance they give to each of the factors/inputs through the weights that they have provided, *d.)* how the things compared (in this case, the study drug and the standard drug) fare given *a, b* and *c*; and *e.)* a global perspective of what *a, b, c,* and *d* amount to, i.e., through the total utility value.

In the risk-benefit literature in research ethics, we find statements that such an algorithm is undesirable because it “yields one and only one verdict about the risk-benefit profile of each possible protocol” [11]. On this issue, CMHP’s *Reflection* is instructive. The scores in quantitative evaluations are valuable not because of some absolute value, but because these scores can

"…focus the discussion by highlighting the divergences between the assessors and stakeholders concerning choice for weights. The benefit of such analysis methods is that the degree and nature of these divergences can be assessed, even in advance of any compound’s review. The same method might be used with the weights (e.g., of different stakeholders) and make both the differences and the consequences of those differences more explicit. If the analyses agree, decision-makers can be more comfortable with a decision. If the analyses disagree, exact sources of the differences in view will be identified, and this will help focus the discussion on those topics [12]."

Thus, the scores are meant to allow the evaluators to know each others’ values, similarities, differences, and divergences. The divergences and differences could aid in focusing the REC discussion and figure out problem areas in a deliberate, transparent, coherent, and less intuitive manner [15].

Risk-benefit analysis and evaluation together constitute *risk-benefit assessment*[4]*.*

Once risks and benefits have been evaluated versus the evaluators’ given criteria, risk evaluation allows evaluators to decide “which risks need treatment and which do not” [6]. In decision studies, amplifying benefits and modifying risks are possible only after a global understanding of it through risk assessment has been achieved. Thus, after risk-benefit assessment comes *risk treatment.* By risk treatment, we refer to the “process of selection and implementation of measures to modify risk…measures may include avoiding, optimizing, transferring, or retaining risk” [4]. In terms of trials, risk treatment would refer to enhancing the trial’s social value, reducing the risks to the participants, and enhancing the participants’ benefits [11]. There may be concerns especially from REC members who have been used to minimizing risk immediately after its identification that this process necessitates them to suspend such move until risk evaluation is done, a procedure that may be counter-intuitive for some. However, the process of “immediately cutting the risks” also have passed through the process of evaluation, although intuitively and implicitly. An REC member who says that the risks of a certain procedure may be minimized or that the risks are unnecessary given the research question has already implicitly gone through a personal evaluation of what is and what is not necessary in such a clinical trial.

After investigating on the possibilities to modify risk and amplify the benefits, the decision makers would then have to finally decide whether the risks of the trial are justified given the benefits. By *decision making*, we refer to the final discussion of the REC on whether benefits truly outweigh risks, i.e., given all the information provided, are the risks of the trial ethically acceptable due to the merits of the probable benefits?

It is important to note that in the risk literature [4,13], the CHMP *Reflection*[12], and the CIOMS report [16], the risk-benefit tasks are assumed to be done interdependently and that the tasks are reflective of various values, interests, and ethical perspectives. At least for marketing authorization and marketed drug evaluation purposes, the sponsor and/or the investigator are assumed to be responsible for risk-benefit assessment and to a certain extent, the proposal of risk treatment measures. It makes a lot of sense that the sponsor ought to be responsible for risk analysis precisely because in this task, “experts on the systems and activities being studied are usually necessary to carry out the analysis” [4]. The regulatory authorities, on the other hand, are expected to provide guidelines for the risk-benefit analysis criteria. They also ought to provide their own version of risk-benefit evaluation to determine areas of divergences and differences, to extensively discuss risk treatment measures and options, and finally to deliberate and decide based on all these inputs.

#### Conflation of the various risk-benefit tasks by the procedure-level approaches

At the most superficial level, we notice that Wendler and Rid used the terms “risk-benefit assessment” and “risk-benefit evaluation” interchangeably to refer to the one and the same Net Risk Test [11,17]. Nevertheless, it could be argued that this is just a matter of misuse of terms, and that such does not substantially affect the approach that is proposed. Thus, we would need to look deeper into the Net Risk Test to justify our claim that it conflates the various risk-benefit tasks.

In the latest seven-step framework of the Net Risk Test, what ought to be a framework for risk-benefit evaluation of RECs ended up incorporating aspects of risk-benefit assessment, risk treatment, and decision making. The first step, that is, ensuring and enhancing the study’s social value, is risk treatment. The second step, that is, identifying the research interventions, is risk analysis. The third and fourth steps, which are the evaluation and reduction of risks to participants, and the evaluation and enhancing of potential benefits to participants, both fall into risk-benefit evaluation and risk treatment. It is worthwhile to note that in the Net Risk Test, the evaluation and the treatment of risks and benefits were not preceded by the identification of these risks and benefits; instead, prior to the third and fourth steps is the step to identify research interventions, a necessary but incomplete step in risk-benefit analysis. The fifth step, that is, the evaluation whether the interventions pose net risks, is risk-benefit evaluation. The sixth step, which is to evaluate whether the net risks are justified by the potential benefits of other interventions, is decision making. The last step, which is to evaluate whether the remaining net risks are justified by the study’s social value, is also decision making. Thus, the Net Risk Test in principle encompasses all the risk-benefit tasks without taking into account the distinctions, the chronological order among the various tasks, nor the division of labor in the various risk-benefit tasks.

The Component Analysis, just like the Net Risk Test, does the same conflation. In the process of distinguishing procedures into either therapeutic or non-therapeutic, the REC members would first need to identify the procedures to assess, i.e., risk analysis. The REC members would then need to evaluate therapeutic procedures differently compared to non-therapeutic procedures. Therapeutic procedures have to be evaluated on whether clinical equipoise exists, and whether the procedure is consistent with competent care. These two criteria may be considered as ethical principles that ought to be present in the deliberation towards decision making. Thus, these are decision making tasks. Next, the REC members would need to determine if the therapeutic procedure is reasonable in relation to the potential benefits to subjects. Since REC members need to answer questions of “reasonability,” this is a decision making task that presupposes risk-benefit evaluation. Non-therapeutic procedures, on the other hand, would necessitate the assessor to evaluate if risks are minimized and if risks are consistent with sound scientific design. This is risk treatment. Next, the assessor would need to verify if the risk of the non-therapeutic procedure is reasonable in relation to knowledge to be gained. Again, this is a decision making task that presupposes risk-benefit evaluation. In cases where vulnerable patients are involved, the REC members would need to verify if no more than minor increase over minimal risk is involved; this is a discussion that is likely to be present in the deliberation towards decision making, which also presupposes risk-benefit evaluation. Lastly, the assessor would need to make a decision if both therapeutic and non-therapeutic procedures pass. This is decision making. Hence, again, what we have is a system that touches on each of the risk-benefit tasks without making a distinction among the various tasks.

Since the risk-benefit tasks are conflated, the various tasks are necessarily simplified and confused. We have seen that the various risk-benefit tasks are resource intensive (since various experts must be involved), necessarily complex (since a drug trial is rarely simple), and time consuming. This is the reason why they are done separately. To conflate the various tasks into one system that ought to be accomplished within the few hours that the REC convenes is an impossibility. Precisely because of this conflation, plus the consideration that all the risk-benefit tasks ought to be done within the time restrictions of an REC, both procedure-level approaches cursorily and confusedly “accomplish” the various tasks. As such, we cannot expect the procedure-level approaches to have the same level of robustness, transparency, explicitness, and coherence as the various approaches of decision studies have. Neither of the procedure-level approaches could have the same robustness that the value-tree had, for example, in expressing and illustrating the relations between the nature, cause, consequences, as well as the uncertainties, of both risk and benefit components. Neither is also transparent, explicit, and rigorous enough to capture the acceptable risk definitions and the various weights and scores that are reflective of the various values and ethical dispositions that the MAUT method provided. The two procedure-level approaches simply do not require evaluators to be explicit in terms of their evaluative values. Though risk treatment is largely present in both procedure-level approaches, risk treatment, at least in the Net Risk Test, is sometimes confounded with risk evaluation. In the procedure-level approaches, RECs would also not have the benefit of systematically focusing the discussion on divergences and differences that a good risk evaluation makes possible. Lastly, because of the conflation and confusion of the various risk-benefit tasks, REC members are left to their own devices and intuition to decide on what is important to discuss and which is not, and eventually, to decide if the risks are justifiable relative to the benefits. Such a “procedure” could be categorized as a “taking into account and bearing in mind” process, a process that Dowie rightfully criticized as vague, general, and plainly intuitive [15].

## Recommendations

We have seen that the methods from decision studies are more robust, transparent, and coherent than any of the procedure-level approaches. This is not surprising considering the fact that decision studies have been utilized in many various fields for quite some time now. The robustness of the decision studies methods stems from the clear distinction between risk-benefit analysis, risk-benefit evaluation, risk treatment, and decision making. In decision studies, each of the risk-benefit tasks is a system in itself that ought not to be conflated. In addition, in contrast to “taking into account and bearing in mind” processes, decision studies encourage the exposure of beliefs and values [15] precisely because it is from this explicitness that discussions can be defined and ordered. As such, we recommend the following:

a. RECs should make clear what their task is. RECs do not have the time and are not in the best position to do risk analyses. As such, risk analysis must be a task for the sponsor. As regards risk evaluation, RECs ought to provide their own risk-benefit evaluation to pair with the sponsor’s/investigator’s evaluation since this is the best way to systematically point out areas of divergence/convergence. These areas would aid in putting order in REC discussions. The evaluation of risk treatment suggestions and possibly coming up with a revised or different risk treatment appraisal ought to also form part of REC discussions. Lastly, it is obviously the REC’s task to make the final decision on whether the risks of the trial are justified given the benefits.

b. Precisely because such a clarification of tasks is so essential if the REC is to function efficiently, RECs must look into how decision studies may be incorporated in its risk-benefit tasks. This is something we will do in our next article. For now, it is imperative to lay the theoretical groundwork for the urgency of such incorporation.

c. The procedure-level approaches emphasize on the role of the various ethical concepts such as net risk, minimum risk, clinical equipoise, in the risk-benefit task of RECs. These are legitimate concerns; nevertheless, RECs must know when these concepts play a role in the various risk-benefit tasks. Minimal risk, for example, is a concept that ought to be present in risk treatment and/or deliberation towards final decision making.

## Conclusion

Both the Net Risk Test and the Component Analysis conflate risk-benefit analysis, risk-benefit evaluation, risk treatment, and decision making. This makes the risk-benefit task of RECs confusing, if not impossible. It is necessary to make a distinction between these four different tasks if RECs are to be clear about what their task truly is. By looking at decision studies, we realize that RECs ought to evaluate risks and benefits, appraise risk treatment suggestions, and make the final decision. Further clarification and elaboration of these tasks would necessitate research ethicists to look beyond the procedure-level approaches. It further requires research ethicists to allow decision studies discourses into the current discussion on the risk-benefit tasks of RECs. Admittedly, this would take a lot of time and research effort. Nevertheless, the discussion on the REC’s risk-benefit task would be more fruitful and democratic if research ethics opens its doors to other disciplines that could truly help clarify risk-benefit task distinctions.

## Competing Interests

RB’s PhD project is funded by the Dutch Top Institute Pharma. JR works for and holds stocks in GlaxoSmithKline. JvD and GvT have no competing interests to declare.

## Authors’ contributions

All authors were involved in the design of the manuscript. RB did the research and wrote the draft and final manuscript; GvT commented on the drafts, wrote parts of the manuscript, and approved the final version; JR commented on the drafts and approved the final version of the manuscript; JvD commented on the drafts and approved the final version of the manuscript. All authors read and approved the final manuscript.

## Pre-publication history

The pre-publication history for this paper can be accessed here:

http://www.biomedcentral.com/1472-6939/13/6/prepub
